# Integrative Multi-Omics Reveals Microbiome and Genome Streamlining Underlie Ecological Divergence in Chinese and Xinjiang *Cordyceps*: A Preliminary Study

**DOI:** 10.3390/ijms27125241

**Published:** 2026-06-10

**Authors:** Yanpeng Ding, Tongyao Liu, Shengting Guo, Jieying Zhu, Jing Zhu, Qiyong Tang, Qiong Jia, Jianlong Li, Zhidong Zhang, Xiaojing Liu

**Affiliations:** 1 Institute of Microbiology, Xinjiang Academy of Agricultural Sciences/Xinjiang Key Laboratory of Special Environmental Microbiology/National Collection of Microbial Resource for Fertilizer (Xinjiang), Urumqi 830091, China; 2 State Key Laboratory of Medicinal Chemical Biology, Key Laboratory of Molecular Microbiology and Technology of the Ministry of Education, Department of Microbiology, College of Life Sciences, Nankai University, 94 Weijin Road, Tianjin 300071, China; 3 College of Veterinary Medicine, Xinjiang Agricultural University, Urumqi 830052, China

**Keywords:** *Paraisaria gracilis*, *Ophiocordyceps sinensis*, host-microbe coadaptation, ecological divergence, multi-omics, holobiont

## Abstract

Chinese *Cordyceps* (*Ophiocordyceps sinensis*) and Xinjiang *Cordyceps* (*Paraisaria gracilis*) are related entomopathogenic fungi that occupy different elevations and habitats. Whether their holobiont architectures have diverged accordingly is unknown. In this hypothesis-generating study based on samples from single locations (Altai Mountains for Xinjiang *Cordyceps* and Nagqu, Tibet for Chinese *Cordyceps*), we compared the two species using amplicon sequencing, untargeted metabolomics, and comparative genomics. Chinese *Cordyceps* from the sampled site comprises a specialized parasitic fungus and host-adapted bacteria for nutrient acquisition. Xinjiang *Cordyceps* from the Altai site contains diverse saprotrophic fungi and a rhizosphere-like bacterial consortium enriched in oxidative defense and biofilm genes, a finding that may explain why its sclerotia remain intact for 3–5 years in this population. Metabolomic profiles distinguish the two species at these sites. Xinjiang *Cordyceps* shows upregulation of tyrosine and porphyrin pathways, and its bacterial community shows functional enrichment in the same pathways, suggesting cross-kingdom coordination. *P. gracilis* has lost many gene families, and the retained species-specific genes are linked to cell adhesion and acyltransferase activity. Xinjiang *Cordyceps* is not a simple substitute for Chinese *Cordyceps* but appears to represent a different ecological strategy shaped by genome streamlining and host–microbe coadaptation. Our findings generate testable hypotheses for future large-scale, multi-population investigations.

## 1. Introduction

*Cordyceps* is a group of entomopathogenic ascomycetes that parasitize insect larvae or pupae to form fungus–insect complexes [1]. The genus once included over 750 species under Clavicipitaceae, but has since been reclassified into Cordycipitaceae, Ophiocordycipitaceae and Clavicipitaceae [2,3]. These complexes have long been used in traditional food and medicine, with reported benefits for kidney, liver, respiratory, nerve, and heart health, as well as anti-cancer, anti-aging and anti-hyperlipidemic effects [4,5]. Among them, Chinese *Cordyceps*, formed by *Ophiocordyceps sinensis*, is the most well-known species. It grows only in high-altitude regions of the Tibetan Plateau and adjacent areas in China, at 3500 to 5000 m (Qinghai, Gansu, Sichuan and Yunnan provinces). Its narrow distribution, complex infection cycle, and overharvesting have led to a severe resource crisis [6,7,8,9].

Research attention has recently expanded to other *Cordyceps* species with similar bioactive profiles. Xinjiang *Cordyceps*, formed by *P. gracilis* (which also parasitizes *Hepialus* larvae), is a promising candidate. This species is widespread across Eurasia and the Americas but remains understudied. In China, Xinjiang is a major distribution area [5,8,10]. It has long been used as a folk food among Kazakh and Mongolian communities to tonify essence, resolve phlegm, and nourish the lungs and kidneys to treat chronic cough and asthma [11]. Modern studies confirm that Xinjiang *Cordyceps* contains bioactive components such as D-mannitol, polysaccharides, adenosine and amino acids, some at levels comparable to or higher than those in Chinese *Cordyceps*. Recognized for its similar chemical and nutritional properties, it was officially listed in the Drug Standards of Xinjiang Uygur Autonomous Region as a substitute for Chinese *Cordyceps* [12,13]. Importantly, Chinese and Xinjiang *Cordyceps* live in different environments. Chinese *Cordyceps* inhabits alpine meadows above 3000 m, while Xinjiang *Cordyceps* grows in coniferous forests at 1000 to 2000 m. The sclerotia of Xinjiang *Cordyceps* can stay intact underground for 3 to 5 years without rotting, a trait not seen in the easily perishable sclerotia of Chinese *Cordyceps* [5,14]. This difference in durability suggests fundamental divergence in their microbial associations and metabolic profiles.

The biology of *Cordyceps* is not determined by the fungal host alone. The associated microbiome and the collective metabolism of the holobiont play critical roles. In Chinese *Cordyceps*, resident microbes contribute to secondary metabolite production and modulate host interactions [15,16,17]. Multi-omics studies have helped understand this complex micro-ecosystem. However, similar in-depth investigations are lacking for Xinjiang *Cordyceps* and existing work is mostly limited to morphology, basic chemistry, or in vitro assays [5,10,12]. Some metabolic profiling has noted compositional differences between the two species [9,13,18], but whether and how these differences are linked to their distinct microbiomes remains unclear. Comparative analyses of microbiome structure and function between these phylogenetically close yet ecologically distinct species are rare.

In this study, we compared Xinjiang *Cordyceps* (Altai Mountains, Xinjiang) and Chinese *Cordyceps* (Nagqu, Tibet) using an integrated multi-omics strategy. Our primary aim was to test whether these two species, despite being phylogenetically related, exhibit detectable divergence in holobiont architecture when sampled from typical natural habitats. Recognizing the single-site sampling limitation, this study is intended to generate hypotheses for future multi-population validation. Collectively, this study provides a preliminary multi-layered perspective for evaluating Xinjiang *Cordyceps* as a functional food resource, informing its future conservation, sustainable utilization and product development.

## 2. Results

### 2.1. Comparative Analysis of Fungal Communities

**Figure 1:**
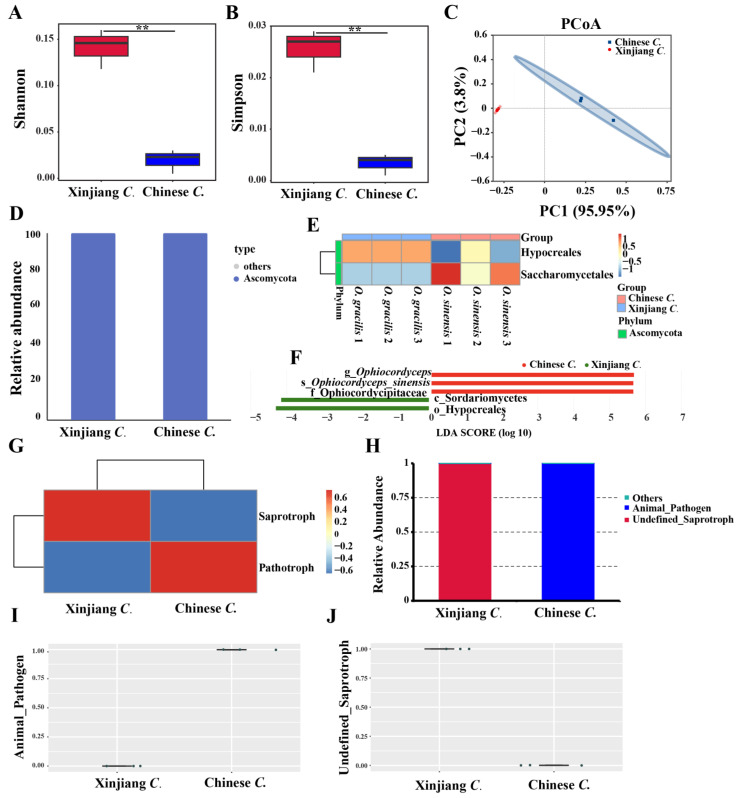
Fungal community diversity and structure of Xinjiang and Chinese *Cordyceps* (*n* = 3). (**A**,**B**) Alpha diversity analysis based on Shannon (**A**) and Simpson (**B**) indices. (**C**) Principal Coordinate Analysis (PCoA) based on unweighted UniFrac distances. (**D**,**E**) Relative abundance at phylum (**D**) and genus (**E**) levels. (**F**) Linear Discriminant Analysis Effect Size (LEfSe) analysis showing differentially abundant fungal taxa. (**G**,**H**) Trophic modes (**G**) and functional guilds (**H**) of the two *Cordyceps*. (**I**) Comparison of the Animal Pathogen guild. (**J**) Comparison of the Undefined Saprotroph guild. ** *p* < 0.01, Student’s *t*-test.

The samples of Xinjiang and Chinese *Cordyceps* were analyzed by amplicon sequencing. All samples analyzed in this study were collected from a single geographical location per species (Altai Mountains for Xinjiang *Cordyceps*; Nagqu, Tibet for Chinese *Cordyceps*), with three biological replicates each. After filtering out samples with a total sequence count below 100, a total of 16 fungal and 3894 bacterial amplicon sequence variants (ASVs) were obtained (Supplementary Files S2 and S3). Alpha diversity indices (Shannon, Simpson, Chao1, observed features) were higher in Xinjiang *Cordyceps*, though differences in Chao1 and observed features were not significant (Figure 1A,B and Figure S1A,B). Beta diversity analysis using Principal Coordinate Analysis (PCoA) and Non-metric Multidimensional Scaling (NMDS) revealed clear separation between the two fungal communities (Figure 1C and Figure S1C). Fungal community structures differed markedly between the two species. Ascomycota dominated both (Figure 1D,E), but Hypocreales composition was more diverse in Xinjiang *Cordyceps*, with several ASVs unassignable to known families or genera. Linear Discriminant Analysis Effect Size (LEfSe) analysis identified the order Hypocreales and associated unclassified families as the most significant biomarkers for Xinjiang *Cordyceps* (Figure 1F).

Functional prediction using FUNGuild revealed marked differences in trophic modes and functional guilds between the two fungal communities. The Xinjiang *Cordyceps* mycobiome was characterized by a high proportion of saprotrophic fungi, whereas pathotrophic fungi were significantly more abundant in Chinese *Cordyceps*. (Figure 1G,H). Accordingly, the guild “Animal Pathogen” was enriched in Chinese *Cordyceps*, consistent with its role as an insect parasite (Figure 1I). In contrast, the guild “Undefined Saprotroph” was predominantly associated with Xinjiang *Cordyceps* (Figure 1J). These results indicate that the compositional divergence of the fungal communities translates into distinct ecological functional potentials, highlighting a distinct fungal assemblage in Xinjiang *Cordyceps* enriched with less common and taxonomically unclassified lineages.

### 2.2. Comparative Analysis of Bacterial Communities

**Figure 2:**
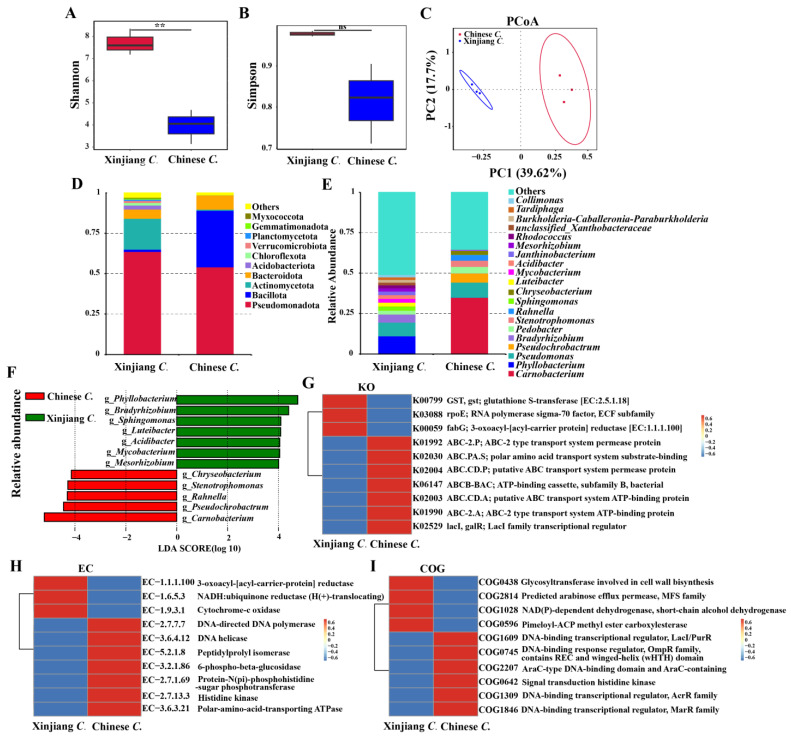
Bacterial community diversity and structure of Xinjiang and Chinese *Cordyceps* (*n* = 3). (**A**,**B**) Alpha diversity analysis based on Shannon (**A**) and Simpson (**B**) indices. (**C**) Principal Coordinate Analysis (PCoA) based on unweighted UniFrac distances. (**D**,**E**) Relative abundance at phylum (**D**) and genus (**E**) levels. (**F**) LEfSe analysis showing differentially abundant bacterial taxa. (**G**) Functional prediction at the KO level. (**H**) Functional prediction at the EC level. (**I**) Functional prediction at the COG level. ** *p* < 0.01, Student’s *t*-test.

Bacterial communities differed significantly between the two *Cordyceps* species. Alpha diversity indices (Shannon, Simpson, Chao1, observed features) were higher in Xinjiang *Cordyceps*, though differences were not significant (Figure 2A,B and Figure S2A,B). Beta diversity analysis based on PCoA and NMDS showed distinct clustering by species (Figure 2C and Figure S2C). At the phylum level, both species were dominated by Pseudomonadota and Bacteroidota (Figure 2D), whereas Bacillota was notably enriched in Chinese *Cordyceps*. At the genus level, Xinjiang *Cordyceps* exhibited a more diverse assemblage, including *Phyllobacterium*, *Pseudomonas*, *Mesorhizobium*, *Bradyrhizobium*, *Sphingomonas*, *Pedobacter*, *Luteibacter*, *Acidibacter* and *Janthinobacterium* (Figure 2E). In contrast, *Carnobacterium*, *Pseudomonas* and *Pseudochrobactrum* were predominantly associated with Chinese *Cordyceps*. LEfSe analysis identified key discriminatory genera (Figure 2F). Biomarkers for Xinjiang *Cordyceps* included *Phyllobacterium*, *Bradyrhizobium*, *Sphingomonas*, *Luteibacter*, *Acidibacter*, *Mycobacterium*, and *Mesorhizobium*, all known for nitrogen cycling, plant growth promotion and degradation of complex organic compounds, and typically associated with soil or rhizosphere environments. Biomarkers for Chinese *Cordyceps* included *Carnobacterium*, *Pseudochrobactrum*, *Rahnella*, *Stenotrophomonas*, and *Chryseobacterium*, which form a consortium with different ecological connotations, including associations with animal hosts or specific environmental niches.

PICRUSt2 functional prediction was performed to infer the metabolic potential of the bacterial communities. Principal coordinate analysis (PCA) based on KO abundances separated the two *Cordyceps* (Figure S2D), indicating divergent functional gene repertoires. At the KO level, Xinjiang *Cordyceps* showed higher abundances of genes related to stress adaptation and metabolic flexibility, including glutathione S-transferase (K00799, gst), ECF subfamily RNA polymerase sigma factor RpoE (K03088), and 3-oxoacyl-[acyl-carrier protein] reductase (K00059, fabG) (Figure 2G). These genes are associated with oxidative defense, envelope stress sensing, and membrane remodeling. At the EC level, Xinjiang *Cordyceps* was significantly enriched in energy metabolism enzymes (NADH:ubiquinone reductase, cytochrome-c oxidase). Chinese *Cordyceps* showed higher levels of EC categories related to DNA replication and protein folding (DNA polymerase, DNA helicase, peptidylprolyl isomerase), carbohydrate utilization (6-phospho-beta-glucosidase, sugar phosphotransferase system), and amino acid transport (polar-amino-acid-transporting ATPase) (Figure 2H). At the COG level, Xinjiang *Cordyceps* displayed significant enrichment in glycosyltransferase (COG0438), a predicted MFS arabinose efflux permease (COG2814), and an NAD(P)-dependent dehydrogenase (COG1028) (Figure 2I). These findings indicate potential for biofilm formation, carbohydrate efflux, and redox homeostasis.

### 2.3. Metabolic Variations Between Xinjiang and Chinese Cordyceps

**Figure 3:**
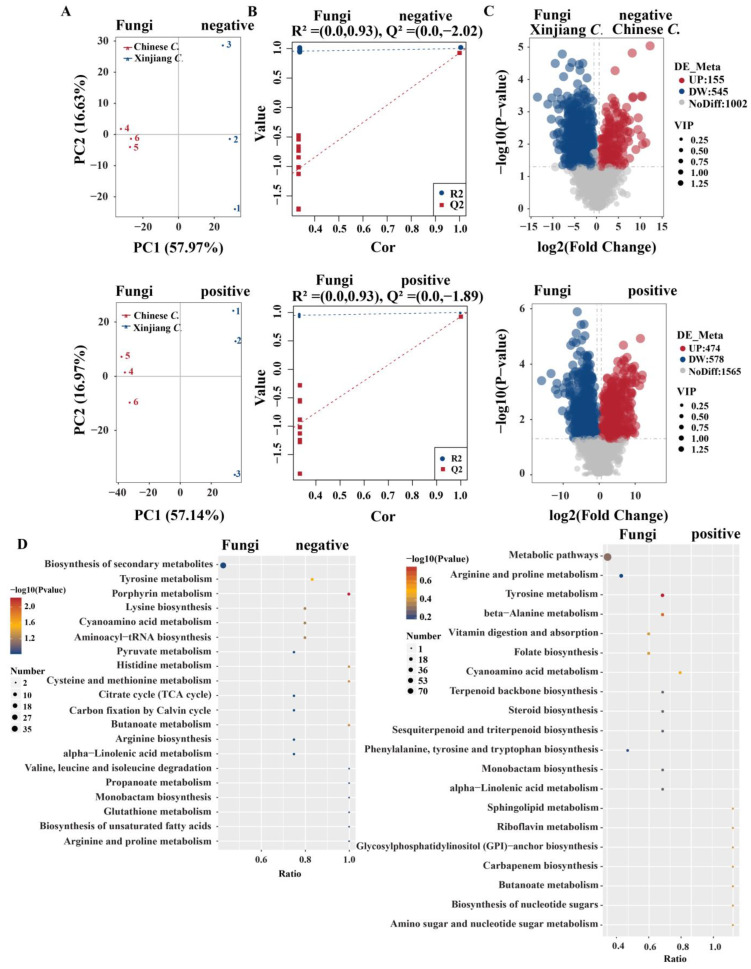
Metabolomic profiling of fungal samples (*n* = 3). (**A**) Principal component analysis (PCA) score plot showing the clustering of fungal samples. (**B**) Orthogonal partial least squares discriminant analysis (OPLS-DA) score plot confirming group separation. (**C**) Bar plot analysis of differentially expressed metabolites, illustrating the marked upregulation of glycerophospholipids and plant secondary metabolites in the two *Cordyceps*. (**D**) KEGG pathway enrichment analysis of differential metabolites, identifying tyrosine metabolism and porphyrin metabolism as significantly enriched pathways.

**Figure 4:**
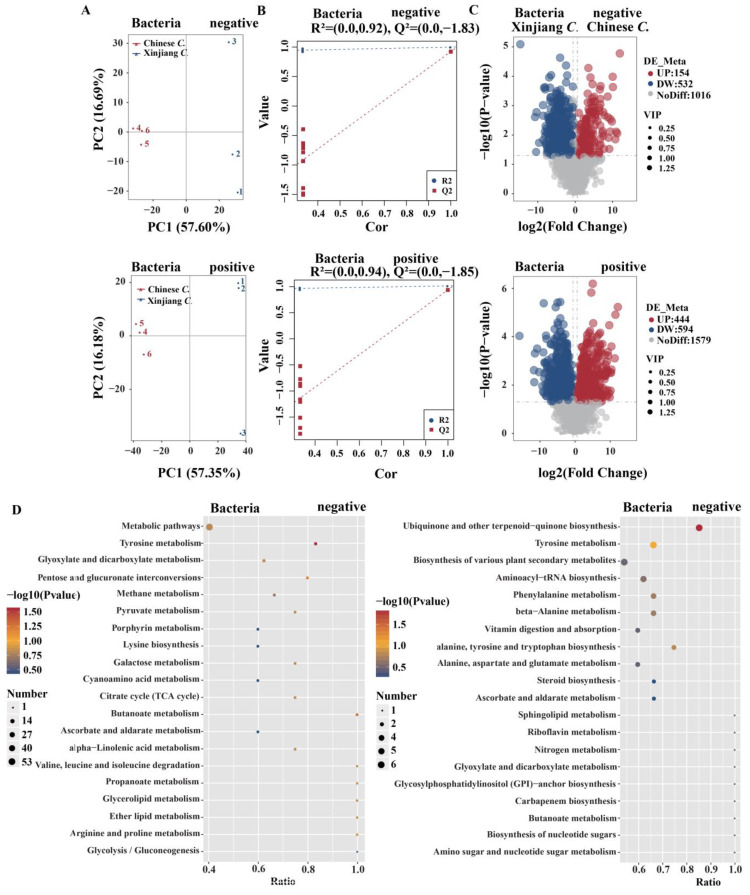
Metabolomic profiling of bacterial samples (*n* = 3). (**A**) Principal component analysis (PCA) score plot showing the clustering of fungal samples. (**B**) Orthogonal partial least squares discriminant analysis (OPLS-DA) score plot confirming group separation. (**C**) Bar plot analysis of differentially expressed metabolites, illustrating the marked upregulation of glycerophospholipids and plant secondary metabolites in the two *Cordyceps*. (**D**) KEGG pathway enrichment analysis of differential metabolites, identifying tyrosine metabolism and porphyrin metabolism as significantly enriched pathways.

Untargeted metabolomics was performed to compare the metabolic profiles of the two *Cordyceps* species. Quality control (QC) samples clustered tightly in the PCA score plots, indicating good system stability and data reproducibility (Figure S3). A total of 4319 metabolites were annotated, with 2617 in positive ion mode and 1702 in negative ion mode. PCA and orthogonal partial least squares discriminant analysis (OPLS-DA) showed clear separation between the two species (Figure 3A,B and Figure 4A,B). The OPLS-DA model exhibited strong predictive power (R^2^Y = 0.996, Q^2^ = 0.977), confirming distinct metabolic profiles.

Differential metabolite analysis identified 474 upregulated and 578 downregulated metabolites in positive ion mode, and 155 upregulated and 545 downregulated in negative ion mode in Xinjiang *Cordyceps* relative to Chinese *Cordyceps* (Figure 3C and Figure 4C). Compared to the latter, the former exhibited markedly higher levels of metabolites belonging to the glycerophospholipid (PC, PE, PS) and plant secondary metabolite (flavonoids/isoflavonoids, terpenoids and glycosides, alkaloids) classes (Figure S4). Notably, KEGG pathway enrichment analysis showed that the differentially expressed metabolites were significantly enriched only in tyrosine metabolism, which provides intermediates for the TCA cycle, and porphyrin metabolism essential for mitochondrial respiratory chain function. These pathways are closely associated with antioxidant activity and cellular energy metabolism (Figure 3D and Figure 4D). Thus, despite their pronounced changes, the majority of glycerophospholipids and plant secondary metabolites did not correspond to significantly enriched KEGG pathways.

### 2.4. Integrative Analysis of Microbiome and Metabolome

**Figure 5:**
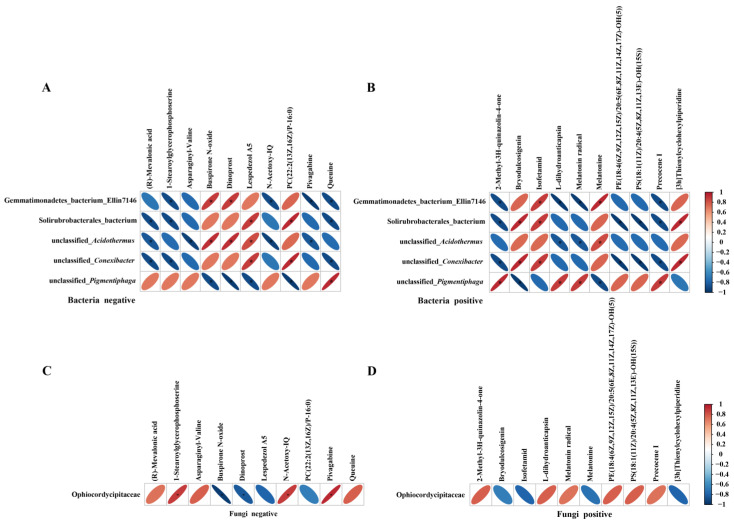
Integrative analysis of microbiome and metabolome correlations (*n* = 3). (**A**,**B**) Bacterial taxa with metabolites detected in negative ion mode (**A**) and positive ion mode (**B**) correlations. (**C**,**D**) Fungal taxa with metabolites detected in negative ion mode (**C**) and positive ion mode (**D**) correlations. The asterisk (*) represents statistical significance in the correlation analysis.

To explore potential associations between microbial taxa and metabolite profiles, Spearman rank correlation analysis was performed on differential microbial lineages and metabolites (VIP > 1.0, *p* < 0.05). Correlations with |ρ| > 0.6 and *p* < 0.05 were considered significant. Several undercharacterized bacterial lineages enriched in Xinjiang *Cordyceps* exhibited strong positive correlations with multiple bioactive metabolites. These included unclassified *Pigmentiphaga*, unclassified *Conexibacter*, unclassified *Acidothermus*, Solirubrobacterales bacterium, and Gemmatimonadetes bacterium Ellin7146 (Figure 5A,B). Metabolites positively correlated with these taxa included buspirone N-oxide, dinoprost, lespedezol A5, PC(22:2(13Z,16Z)/P-16:0), queuine, melatonin, melatonin radical, L-dihydroanticapsin, isofetamid, bryodulcosigenin, 2-methyl-3H-quinazolin-4-one, [^3^H]thienylcyclohexylpiperidine, and precocene I. For fungal taxa, analysis was performed at the family level due to limited taxonomic resolution of Xinjiang *Cordyceps*-associated fungi, which were primarily classified within Ophiocordycipitaceae. This family displayed significant positive correlations with three metabolites, including 1-stearoylglycerophosphoserine, N-acetoxy-IQ, and pivagabine (Figure 5C,D). These correlation networks provide testable hypotheses for microbial contributions to the distinctive metabolic phenotype of this species and establish a foundation for future functional validation.

### 2.5. Comparative Genomic Analysis of P. gracilis and O. sinensis

**Figure 6:**
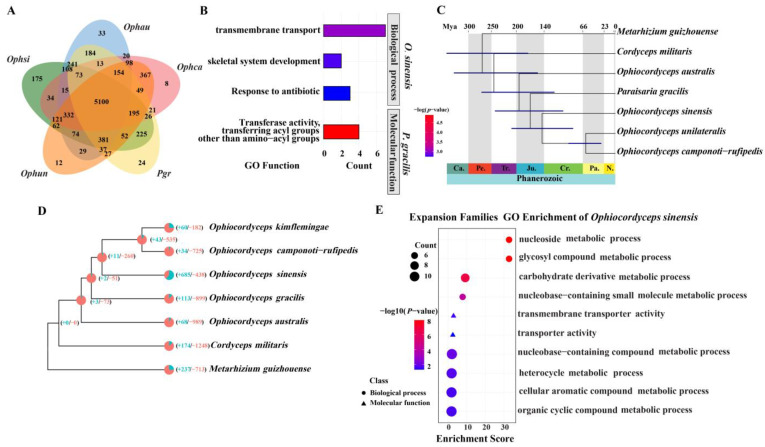
Comparative genomic analysis of Xinjiang and Chinese *Cordyceps*. (**A**) Venn diagram showing shared and unique gene families among five Ophiocordycipitaceae species. (**B**) GO enrichment analysis of species-specific gene families in two *Cordyceps*. (**C**) Phylogenetic tree and divergence time estimation based on single-copy orthologous genes. (**D**) Gene family expansion and contraction dynamics. (**E**) GO enrichment analysis of significantly expanded gene families in Chinese *Cordyceps*.

Comparative genomic analysis provided independent evidence supporting the ecological differentiation inferred from microbiome and metabolome data. Venn diagram analysis of gene family clustering among five *Ophiocordyceps*-like fungi showed that *O. sinensis* contains 7214 gene families, while *P. gracilis* contains 6802 families. The five fungi share 5100 core gene families (Figure 6A). *O. sinensis* possesses the highest number of species-specific families (175 clusters), whereas *P. gracilis* has far fewer, with only 24 clusters. GO enrichment analysis indicated that species-specific genes in *O. sinensis* are primarily enriched in functions related to membrane transport, multicellular organism processes, and antibiotic synthesis, while those in *P. gracilis* are enriched only in acyltransferase activity (Figure 6B). Pairwise comparison revealed that *O. sinensis* and *P. gracilis* share 6286 families, with 315 and 54 species-specific families, respectively (Figure S5A). GO enrichment analysis showed that unique gene families of *P. gracilis* are functionally concentrated in cellular interactions with other organisms, including aggregation and adhesion (Figure S5B). In contrast, unique gene families of *O. sinensis* are enriched in molecular functions including lipase activity and oxidoreductase activity (Figure S5C), as well as biological processes such as amino acid transport, pH regulation, and nucleoside metabolism (Figure S5D).

Phylogenetic analysis based on orthologous single-copy genes placed *P. gracilis* within the same monophyletic group as other *Ophiocordyceps* fungi. The common ancestor of *Ophiocordyceps*-like fungi diverged approximately 169.78 million years ago (95% HPD: 158.276–327.614 Mya, Figure 6C). Gene family contraction and expansion analysis revealed that relative to the ancestral node, the *O. sinensis* genome gained 685 genes and lost 438 genes, with 39 gene families significantly expanded (98 genes) and 1 family contracted (2 genes, *p* < 0.05). The *P. gracilis* genome gained 113 genes and lost 899 genes relative to the ancestral node, with 11 gene families significantly expanded (33 genes) and 3 families contracted (6 genes, *p* < 0.05) (Figure 6D and Table 1). Notably, 10 GO terms were enriched exclusively among genes expanded in *O. sinensis* (Figure 6E).

## 3. Discussion

### 3.1. Microbial Communities Underpin Ecological Divergence of Xinjiang Cordyceps in the Sampled Populations

In the Altai and Nagqu samples analyzed here, the two *Cordyceps* species exhibited fundamentally different biological architectures, suggesting that their divergence in ecology and sclerotial durability may be explained by these microbial differences. These differences may explain their divergence in ecology and sclerotial durability. The differences are present in both fungal and bacterial communities. In Chinese *Cordyceps*, the fungal community is a specialized entomopathogen. It consists of a near-clonal population of *O. sinensis* with host-associated bacteria such as *Carnobacterium* and *Pseudochrobactrum* [19,20,21]. The functional profiles of these communities are biased toward nutrient transport and macromolecular biosynthesis. This configuration fits a commensal lifestyle within a stable parasitic niche [22,23]. This specialization explains why Chinese *Cordyceps* is rare and its sclerotia easily perishable [5,14].

Xinjiang *Cordyceps* shows a different architecture. Its fungal community is dominated by unclassified Hypocreales lineages with predicted saprotrophic functions. This suggests a shift away from obligate dependence on insect hosts. Comparative genomics provides a genetic basis for this shift. The *P. gracilis* genome has lost many gene families relative to its ancestor (899 lost versus 113 gained). Its species-specific genes (24 clusters) are enriched only in acyltransferase activity and cell adhesion. These functions promote microbial interaction and surface colonization, which fits with the close associations we observed between the fungus and its bacterial consortium. The bacterial community of this species reinforces this divergence. It harbors a more diverse consortium dominated by rhizosphere-associated genera including *Phyllobacterium*, *Bradyrhizobium*, and *Sphingomonas* [24,25,26,27]. These taxa are known for nitrogen fixation, plant growth promotion, and secondary metabolite production. Functional predictions showed enrichment of genes involved in oxidative defense, envelope stress sensing, membrane remodeling, biofilm formation, and energy metabolism.

Together, these microbial features are consistent with the most striking trait of Xinjiang *Cordyceps*: its sclerotia can remain intact underground for three to five years without rotting [5,14]. The shift toward saprotrophic fungi reduces dependence on labile host resources. The bacterial consortium contributes protective functions: oxidative defense prolongs tissue integrity, biofilm formation may suppress competing organisms, and metabolic versatility supports a broader range of metabolites. This functional repertoire provides a mechanistic link between the distinct microbiome of this species and its ecological durability.

### 3.2. Genomic Basis for Divergent Host Adaptation Strategies in the Sampled Genomes

Species-specific genes and expanded gene families reflect how fungi adapt to their environments and hosts [28]. In the genomes we analyzed, *O. sinensis* has the highest number of species-specific genes (175 clusters). *O. sinensis* lives in a highly specialized habitat, alpine cold regions at 3500 to 5000 m [29]. After infecting its host *Hepialus xiaojinensis*, it forms a sclerotium during the host’s overwintering period [30]. During this phase, genes for transmembrane transport are strongly upregulated [31,32]. This matches the enrichment of transmembrane transport functions among its species-specific genes. To counter other soil microorganisms, *O. sinensis* also shows capabilities in antibiotic resistance and detoxification, possibly through monooxygenases and oxidoreductases that enhance its competitiveness.

*Ophiocordyceps* fungi generally have strict host specificity. The host specificity of *O. sinensis* is likely linked to its species-specific genes and large-scale gene family expansion. The nucleoside metabolic genes expanded in its genome are mainly involved in cordycepin biosynthesis, a secondary metabolite important for host toxicity. *O. sinensis* requires a long parasitic association with its host. During the process from host weakening to death, it obtains nutrients from host lymph and hemolymph [33,34], and several genes involved in homeostasis are enriched. In contrast, *P. gracilis* has a broader host range within Lepidoptera. Its species-specific genes are enriched in aggregation and cell adhesion functions, which may help it attach quickly to different hosts. Its expanded gene families may contribute to the synthesis of nucleoside-derived secondary metabolites with broad-spectrum host toxicity. This is consistent with our metabolomic finding that Xinjiang *Cordyceps* has higher levels of various alkaloids and phospholipids.

Phylogenetic analysis based on single-copy orthologous genes placed *P. gracilis* within the genus *Ophiocordyceps*, suggesting that the genus as currently defined may be polyphyletic. The common ancestor of these Ophiocordycipitaceae fungi diverged approximately 169.78 million years ago (95% HPD: 158.276 to 327.614 Mya). This was a period when insects were recovering from the end-Permian mass extinction and diversifying across the world in the Jurassic [35,36,37]. The Karamay fossil assemblage in Central Asia indicates that ancestors of holometabolous insects underwent rapid diversification [38], which broadly coincides with the divergence time of the common ancestor of these fungi. The host adaptation strategy of *P. gracilis* may therefore be closer to that of the common ancestor. Its broader host range and microbial partnership may represent ancestral traits, while the extreme specialization of *O. sinensis* is a derived adaptation to high-altitude alpine environments.

### 3.3. Metabolic Signatures of Ecological Divergence and Microbial Synergy in the Sampled Populations

The metabolic divergence between the two *Cordyceps* species provides evidence that their distinct microbial partnerships shape their chemistry. Metabolomic profiling showed complete separation between the two species. Xinjiang *Cordyceps* accumulated higher levels of alkaloids, sphingolipids, phospholipids, and plant-associated compounds. Several of these metabolites were found only in this species or at much higher abundance, suggesting they could serve as chemical markers for species identification within the sampled sites. Two lines of evidence support a mechanistic link between the microbiome and these metabolic differences. First, KEGG enrichment analysis identified two pathways significantly upregulated in Xinjiang *Cordyceps*: tyrosine metabolism and porphyrin metabolism. Both pathways are connected to antioxidant activity, stress resistance, and energy metabolism [39,40,41].

Tyrosine metabolism contributes to energy production through its degradation pathway. Tyrosine is broken down to acetoacetate and fumarate [42,43]. Fumarate enters the TCA cycle, supporting ATP and reducing equivalent generation. Tyrosine metabolism also helps counter oxidative stress. Tyrosine biosynthesis and degradation pathways protect against reactive oxygen species-induced damage [44,45,46]. Tyrosine aminotransferase, the first enzyme in tyrosine degradation, has been shown to enhance antioxidant capacity [47]. The enhanced antioxidant potential of tyrosine-derived metabolites is further supported by the superior aromatic-electron donating aptitude of the tyrosyl sidechain compared to phenylalanyl, which facilitates radical stabilization and redox cycling [48].

Porphyrin metabolism controls heme biosynthesis. Heme is a prosthetic group for cytochromes, catalase, and peroxidase [49,50]. Cytochromes are central to the mitochondrial electron transport chain and oxidative phosphorylation. Cytochrome c oxidase depends on heme a, a specialized heme derivative made through the porphyrin pathway. Without enough heme, mitochondrial respiration is impaired. Heme-containing enzymes like catalase and peroxidase are also primary defenses against oxidative stress [50]. Studies have shown that enhancing porphyrin biosynthesis connects to oxidative stress and energy metabolism [51]. In plants, porphyrin metabolism contributes to maintaining photosynthesis and activating antioxidant responses under stress [52].

Notably, these same pathways were significantly enriched in the PICRUSt2 functional predictions of the bacterial community in Xinjiang *Cordyceps*. This cross-kingdom convergence suggests that the distinctive metabolic phenotype of this species is not solely due to its fungal genome. The bacterial consortium appears to complement or amplify the metabolic capabilities of the fungal host, jointly enhancing energy metabolism and stress resistance. Second, correlation analyses linked specific microbial taxa to the production of bioactive metabolites. Several unclassified Hypocreales fungal families correlated strongly with alkaloid, sphingolipid, and phospholipid derivatives. More compelling were the bacterial correlations. Five undercharacterized lineages, including unclassified *Pigmentiphaga*, *Conexibacter*, *Acidothermus*, Solirubrobacterales bacterium, and Gemmatimonadetes bacterium Ellin7146, showed robust associations with multiple metabolites. These included the antioxidant melatonin, the insect juvenile hormone antagonist precocene I, the mevalonate pathway intermediate (R)-mevalonic acid, and several membrane-active phospholipids [53,54,55]. Several of these metabolites have not been reported before in *Cordyceps* species, indicating that Xinjiang *Cordyceps* contains unexplored chemical diversity. The fact that these metabolites correlate with bacterial lineages, not with the dominant fungus, supports the view that the bacterial consortium directly contributes to the metabolic phenotype [56,57].

A noteworthy observation emerged from comparing the breadth of metabolic changes with the limited number of enriched pathways. Xinjiang *Cordyceps* exhibited markedly higher levels of glycerophospholipids and plant secondary metabolites, including flavonoids, isoflavonoids, terpenoids, glycosides, and alkaloids. However, KEGG enrichment analysis showed that the differentially accumulated metabolites were significantly enriched only in tyrosine metabolism and porphyrin metabolism. This discrepancy warrants further consideration. Several factors may explain why these abundant compound classes did not correspond to significantly enriched KEGG pathways. First, many of these metabolites, particularly plant-associated compounds, are not well represented in current KEGG databases, which are biased toward model organisms and core metabolic pathways [58]. Second, the production of such compounds may involve microbial contributors whose metabolic pathways are not fully captured by fungal-centric annotation systems [59,60]. The strong correlations we observed between bacterial lineages and several bioactive metabolites support this interpretation. Third, these metabolites may represent terminal products or modified derivatives whose biosynthetic precursors and upstream pathways are shared with the enriched pathways, even if the compounds themselves do not map directly to them. The convergence between metabolomic enrichment and bacterial functional predictions provides additional context. Tyrosine metabolism and porphyrin metabolism were both significantly enriched in the PICRUSt2 functional predictions of the Xinjiang *Cordyceps* bacterial consortium. This cross-kingdom alignment suggests that the metabolic features most directly linked to energy production and stress resistance may be amplified by microbial collaboration, while the broader chemical diversity, including plant-like compounds, may arise from specialized microbial taxa acting in concert with the fungal host. These findings reinforce the view that the distinctive metabolic phenotype of Xinjiang *Cordyceps* emerges from the integrated activity of its entire microbial community rather than from the fungal genome alone.

These findings change how we view Xinjiang *Cordyceps*. Instead of assessing it mainly by its similarity to Chinese *Cordyceps*, our data show that its value lies in its unique chemical repertoire shaped by microbial collaboration. This distinction moves the discussion beyond substitution. The identification of microbe metabolite correlations also opens new questions for future work. If specific microbial taxa are consistently linked to particular metabolites, they could serve as indicators for harvest selection or targets for cultivation strategies. Collectively, these results demonstrate that the compositional differences between the two species are not incidental. They are rooted in fundamentally different biological architectures. In Xinjiang *Cordyceps*, a streamlined fungal genome partners with a metabolically versatile bacterial consortium to produce a distinctive chemical profile. This microbial collaboration contributes not only to chemical diversity but also to the exceptional durability of its sclerotia.

### 3.4. Implications for Understanding Host–Microbe Coadaptation: Insights from Single-Site Samples

The distinct biological architecture of Xinjiang *Cordyceps* observed in this study has implications for how we study host–microbe interactions in entomopathogenic fungi. Previous studies using isolated and fermented mycelia have reported similar bioactive profiles between the two species [12,13]. Those approaches, by necessity, exclude the native microbial consortium that accompanies the intact fungus–insect complex. Our findings show that when the complete biological entity including microbial partners is considered, Xinjiang and Chinese *Cordyceps* diverge markedly in both metabolic output and functional potential. The similarity seen in axenic culture reflects only the core fungal metabolism, while the distinctive traits of the natural sclerotia emerge from the integrated activity of the fungal host and its co-associated microbial community.

From an evolutionary perspective, Xinjiang *Cordyceps* shows three complementary features. First, genomic streamlining and loss of parasitic genetic machinery may reduce metabolic regulatory complexity. Second, its metabolically versatile, biofilm-forming bacterial consortium directly contributes to sclerotial durability. Third, its distinctive metabolite repertoire, partly microbial in origin, provides a chemical basis for differentiating it from Chinese *Cordyceps*. Together, these findings position Xinjiang *Cordyceps* not as a substitute for Chinese *Cordyceps* but as a distinct system shaped by genome streamlining and microbial synergy. Beyond its specific findings, this study offers a conceptual connection between multi-omics approaches and the idea of holobiont-level function. Both the concept of a functional microbiota and the holistic view in traditional medicine emphasize that effects arise from synergistic interactions within a complex community rather than from a single agent [61,62]. While prior research has focused on identifying specific compounds in *Cordyceps* species [4,5,12,56], our study aligns with this holistic paradigm by investigating the entire microbial consortium and its collective metabolic output [11].

### 3.5. Limitations and Future Directions

As this is a hypothesis-generating study based on single-site samples with n = 3 per species, we acknowledge that this sample size and geographical coverage are limited for robust statistical inference in microbiome and metabolomics studies. However, the consistent and strong differences observed in this study justify further investigation. The risk of overfitting, particularly in multivariate models such as OPLS-DA, cannot be completely excluded. Therefore, our findings should be interpreted as preliminary and hypothesis-generating, and future studies with larger sample sizes across multiple geographical populations are needed to validate and extend our conclusions. Functional predictions based on marker genes remain inferential and require validation through metagenomics, cultivation, and reconstitution experiments. The five bacterial lineages correlated with multiple bioactive metabolites are priorities for isolation, genome sequencing, and metabolomic profiling. Establishing causal relationships between these microbial taxa and specific metabolite production will require controlled co-culture experiments. The specific contributions of the fungal host versus its microbiome to the observed metabolic phenotype also need further investigation through comparative metabolomics of cultured *P. gracilis* mycelia with and without its native microbial consortium. Answering these questions will clarify whether the distinctive metabolic profile of Xinjiang *Cordyceps* can be reconstituted or enhanced through microbiome manipulation.

## 4. Materials and Methods

### 4.1. Sample Collection

The Xinjiang *Cordyceps* were collected from coniferous forest soil in the Altai Mountains, Xinjiang Uygur Autonomous Region, China. The Chinese *Cordyceps* were collected from an alpine meadow in Nagqu City, Tibet Autonomous Region, China. These samples have been deposited in the Xinjiang Microbiological Culture Collection Center. All samples were stored at −80 °C until analysis.

### 4.2. DNA Extraction and Amplicon Sequencing

Total genomic DNA was extracted from 0.25 g of each homogenized sample using the DNeasy PowerSoil Pro Kit (Qiagen, Hilden, Germany). DNA concentration and purity were assessed with a NanoDrop spectrophotometer (Thermo Fisher Scientific, Waltham, MA, USA) and agarose gel electrophoresis.

The V3-V4 region of the bacterial 16S rRNA gene was amplified with primers 338F (5′-ACTCCTACGGGAGGCAGCAG-3′) and 806R (5′-GGACTACHVGGGTWTCTAAT-3′). The fungal ITS1 region was amplified with primers ITS5-1737F (5′-GGAAGTAAAAGTCGTAACAAGG-3′) and ITS2-2043R (5′-GCTGCGTTCTTCATCGATGC-3′). Each 25 μL PCR contained 12.5 μL of 2×Phusion High-Fidelity PCR Master Mix (New England Biolabs, Ipswich, MA, USA), 0.2 μM of each primer, and ~10 ng of template DNA. Thermal cycling conditions were: 98 °C for 1 min; 30 cycles of 98 °C for 10 s, 50 °C for 30 s, 72 °C for 30 s; and final extension at 72 °C for 5 min. PCR products were purified with Agencourt AMPure XP beads (Beckman Coulter, Brea, CA, USA). Equimolar purified amplicons were pooled for library preparation, and library quality was checked on a Bioanalyzer 2100 system (Agilent Technologies, Santa Clara, CA, USA). Paired-end sequencing (2 × 250 bp) was performed on an Illumina NovaSeq 6000 platform (Novogene, Beijing, China).

Raw reads were processed in QIIME2 (version 2023.5). Primers were trimmed, and reads were quality-filtered, denoised and merged using DADA2 to generate amplicon sequence variants (ASVs). Chimeras were removed with the uchime-denovo method in VSEARCH. Bacterial 16S ASVs were taxonomically assigned against the SILVA database (release 138.1), and fungal ITS ASVs against the UNITE database (version 9.0), both at 97% similarity.

### 4.3. Untargeted Metabolomics Analysis

Freeze-dried and ground sample (50 mg) was extracted with 500 μL of ice-cold 80% methanol containing 0.1% formic acid. The mixture was vortexed, sonicated on ice for 30 min, and centrifuged at 15,000× *g* for 20 min at 4 °C. The supernatant was diluted with LC-MS grade water to 53% methanol and centrifuged again. The final supernatant was filtered through a 0.22 μm membrane for LC-MS/MS analysis.

Chromatographic separation was performed on a Vanquish UHPLC system (Thermo Fisher Scientific, USA) equipped with a Hypersil Gold C18 column (100 mm × 2.1 mm, 1.9 μm) maintained at 40 °C. The mobile phase consisted of (A) 0.1% formic acid in water and (B) methanol. The flow rate was 0.2 mL/min with the following gradient: 2% B (0–1.5 min), linear to 85% B (1.5–4.5 min), to 100% B (4.5–10.5 min), held at 100% B (10.5–11 min), returned to 2% B (11–11.1 min), and re-equilibrated at 2% B (11.1–12 min). Mass spectrometry was performed on an Orbitrap Exploris™ 120 mass spectrometer (Thermo Fisher Scientific, USA) in positive and negative electrospray ionization modes. Spray voltage was set to 3.5 kV (positive) and 3.2 kV (negative); capillary temperature was 320 °C; sheath gas and auxiliary gas were 35 and 10 arbitrary units, respectively.

Raw data were processed with Compound Discoverer™ (version 3.3, Thermo Fisher Scientific) for peak detection, alignment and deconvolution. Metabolites were identified by matching accurate mass and MS/MS spectra against mzCloud, mzVault and MassList databases (mass tolerance 5 ppm). Peak areas were used for quantification. Data were normalized by probabilistic quotient normalization (PQN). Metabolites with a coefficient of variation >30% in quality control (QC) samples were removed. Principal component analysis (PCA) and orthogonal partial least squares discriminant analysis (OPLS-DA) were performed using SIMCA-P (version 16.0.2, Umetrics, Sweden). Differential metabolites were selected with VIP > 1.0, fold change > 1.5 or <0.67, and *p* < 0.05 (Student’s *t*-test). Pathway enrichment analysis was performed using the KEGG database.

### 4.4. Integrated Microbiome-Metabolome Analysis

Spearman rank correlation analysis was performed to assess associations between microbial communities and metabolites. The analysis incorporated the relative abundance of significantly differential bacterial taxa (at the species level) and fungal taxa (at the family level), along with differential metabolites (VIP > 1.0, *p* < 0.05). Correlations with |ρ| > 0.6 and *p* < 0.05 were considered statistically significant and visualized using the pheatmap and corrplot packages in R (version 4.3.1).

### 4.5. Comparative Genomics Analysis

A total of seven genomes were obtained from public databases for the comparative analysis, with their GenBank accession numbers listed in Table 2. Orthologous clusters were identified using OrthoVenn3 [63] from coding genes of five *Ophiocordyceps* species: *O. unilateralis* [64], *O. camponoti-rufipedis* [65], *O. australis* Map64 [65], *O. sinensis* [66], and *Paraisaria gracilis* MB504277 (a synonym of *O. gracilis*) [10]. OrthoFinder [67] was used to cluster orthologous groups among these five species plus *Cordyceps* militaris and *Metarhizium guizhouense*. Single-copy orthologs were selected for phylogenetic tree reconstruction with iqtree2. Divergence times were estimated using MCMCtree in PAML v4.9i [68] under a relaxed molecular clock model (global substitution rate: 10^−9^ substitutions per site per year). Prior calibration (185 million years ago) between *C. militaris* and *M. guizhouense* was obtained from TimeTree 5 [69] and previous studies [70,71]. Gene family expansion and contraction were analyzed with CAFE 5 [72].

### 4.6. Statistical Analysis

Data are presented as mean ± standard error (SE). Comparisons between two groups were performed using unpaired two-tailed Student’s *t*-test. *p* < 0.05 was considered statistically significant. The significance level was set at α = 0.05, and results are presented with 95% confidence intervals for mean differences.

## 5. Conclusions

Based on the single-site samples analyzed in this hypothesis-generating study, the two *Cordyceps* species appear to represent two distinct ecological strategies rather than interchangeable substitutes. Chinese *Cordyceps* from Nagqu is a host specialist whose value comes from rarity and strict dependence on its insect host. In contrast, Xinjiang *Cordyceps* from the Altai Mountains has a streamlined genome and a metabolically versatile, biofilm-forming microbiome. These traits may contribute to its exceptional sclerotial durability and a unique chemical profile. Our work provides the first integrated multi-omics framework for understanding Xinjiang *Cordyceps* as a biologically distinct system shaped by genome streamlining and host–microbe coadaptation. Our findings generate testable hypotheses for future multi-population studies.

## Figures and Tables

**Table 1:** Values at each node in the gene family dynamics analysis and those with *p* < 0.05.

Node	Expansion Genes	Decrease Genes	Remain Genes	Expansion Genes (*p* < 0.05)	Decrease Genes (*p* < 0.05)	Expansion Families (*p* < 0.05)	Decrease Families (*p* < 0.05)
*Cordyceps militaris* (1)	174	1248	5828	41	8	16	4
*Paraisaria gracilis* (2)	113	899	6238	33	6	11	3
*O. sinensis* (3)	685	438	6127	98	2	39	1
*O. camponoti-rufipedis* (4)	34	725	6491	2	6	1	3
*O. unilateralis* (5)	60	182	7008	8	4	4	2
*O.australis* (6)	69	989	6192	14	8	6	4
*Metarhizium guizhouense* (7)	237	713	6300	60	0	20	0
8	Null	Null	Null	Null	Null	Null	Null
9	0	0	7250	0	0	0	0
10	3	73	7174	1	20	1	8
11	2	51	7197	2	2	2	1
12	11	260	6979	5	2	4	1
13	43	535	6672	5	4	2	2

**Table 2:** Species Details and Genome Sources.

Species	Stain	Host	GenBank
*Ophiocordyceps unilateralis*	SC16a	*Camponotus castaneus*	GCA_001272575.2
*Ophiocordyceps camponoti-rufipedis*	Map16	*Camponotus rufipes*	GCA_002591395.1
*Ophiocordyceps australis*	Map64	*Ponerinae sp.*	GCA_002591415.1
*Ophiocordyceps sinensis*	IOZ07	*Hepialus xiaojinensis*	GCA_012934285.1
*Paraisaria gracilis*	MB504277	Lepidoptera	GCA_051529295.1
*Cordyceps militaris*	CM01	Lepidoptera	GCA_000225605.1
*Metarhizium guizhouense*	ARSEF977	Widespread in insects and nematodes	GCA_000814955.1

## Data Availability

The raw sequencing data have been deposited in the NCBI Sequence Read Archive (SRA) under the accession numbers PRJNA1431114 (fungi) and PRJNA1431121 (bacteria). The metabolomics data are available in the OMIX database (PRJCA059241) at the National Genomics Data Center (NGDC), China National Center for Bioinformation/Beijing Institute of Genomics, Chinese Academy of Sciences. The OMIX database is part of the Genome Sequence Archive and is publicly accessible.

## Supplementary Materials

The following supporting information can be downloaded at: https://www.mdpi.com/article/10.3390/ijms27125241/s1.
