# Supplementary material for: Integrative Multi-Omics Reveals Microbiome and Genome Streamlining Underlie Ecological Divergence in Chinese and Xinjiang Cordyceps: A Preliminary Study
Source: Int J Mol Sci. 2026 Jun 10;27(12):5241. doi: 10.3390/ijms27125241 (PMC13299506; doi:10.3390/ijms27125241)
Supplement: Supplementary file 1 [file ijms-27-05241-s001.zip › Supplementary file S1.pdf]

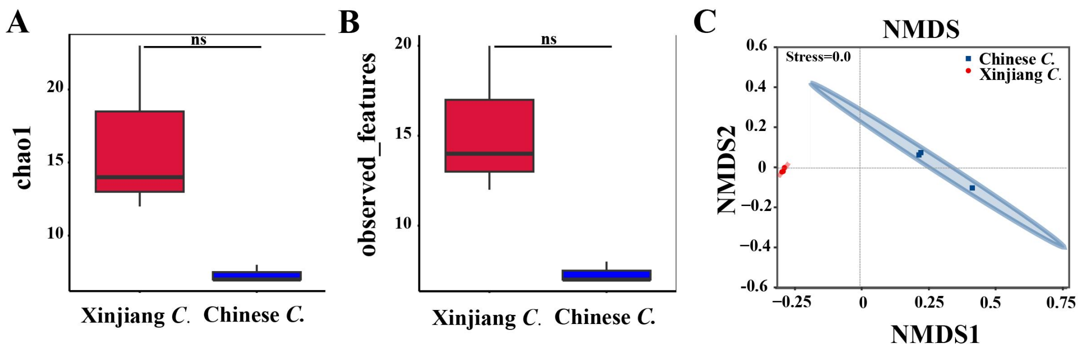

Figure S1. Fungal community diversity and structure of Xinjiang *Cordyceps* and Chinese *Cordyceps* (n = 3). (A, B) Alpha diversity analysis based on chao 1 (A) and observed features (B) indices. (C) Non-metric Multidimensional Scaling (NMDS) based on unweighted UniFrac distances.

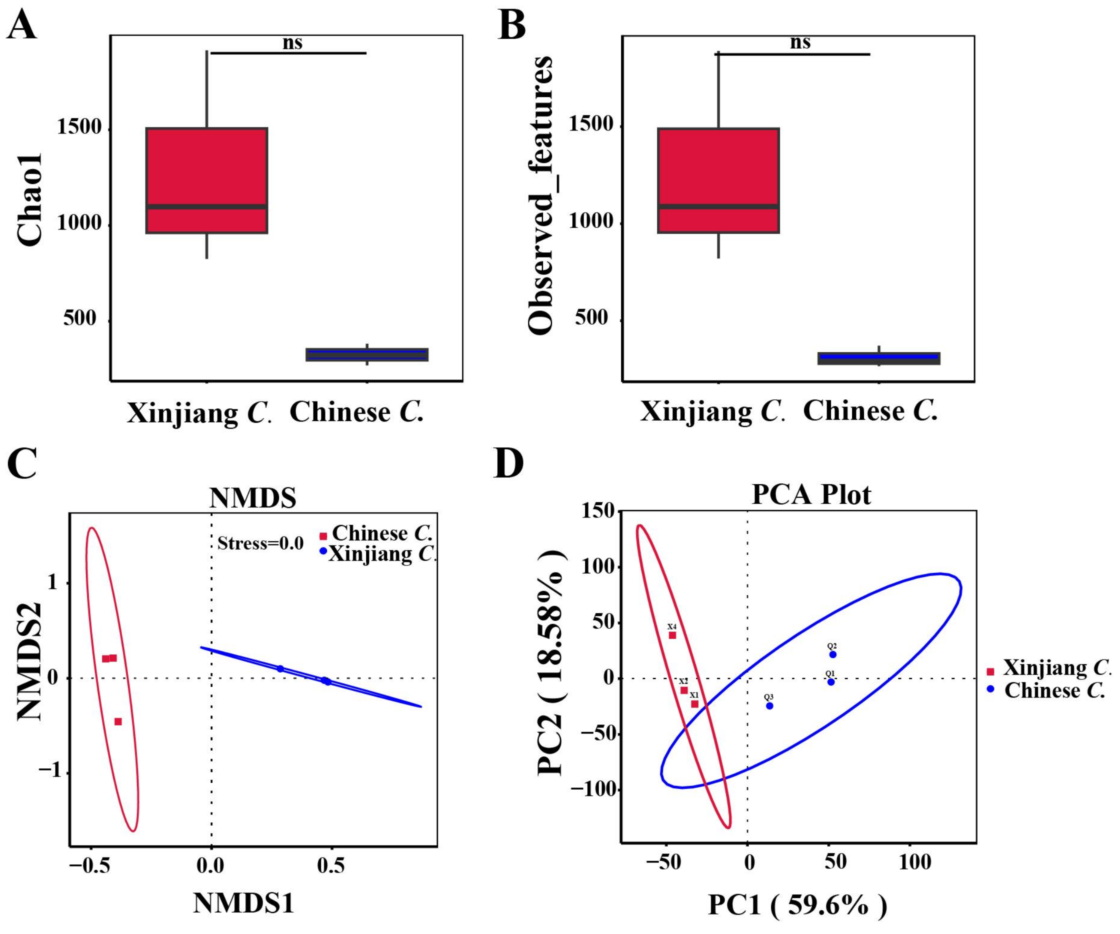

Figure S2. Bacterial community diversity and structure of Xinjiang *Cordyceps* and Chinese

Cordyceps (*n* = 3). (A, B) Alpha diversity analysis based on chao 1 (A) and observed features (B) indices. (C) Non-metric Multidimensional Scaling (NMDS) based on unweighted UniFrac distances.

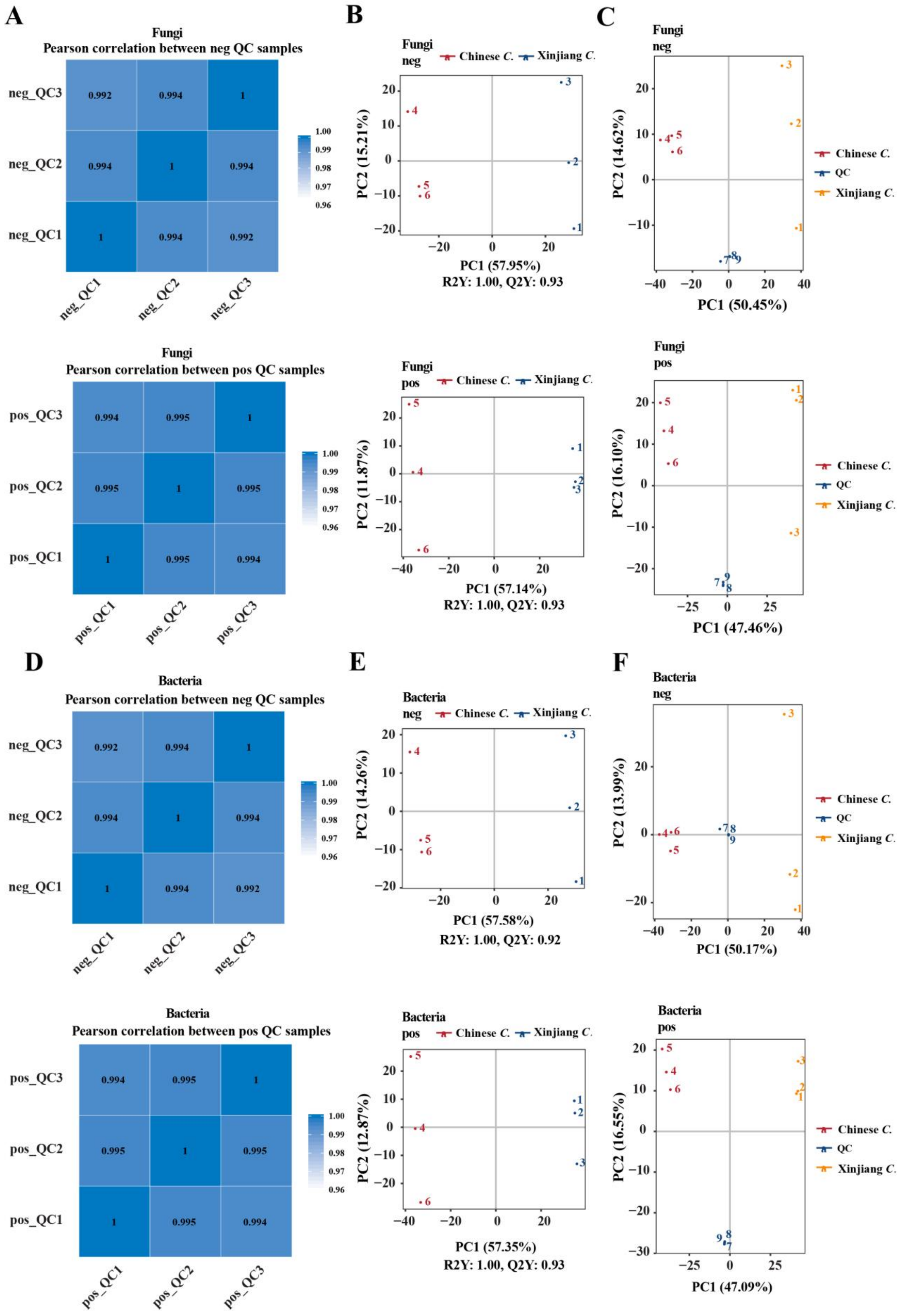

Figure S3. Quality control and reproducibility assessment of untargeted metabolomics data (*n* = 3)**.** (a) Pearson correlation analysis of fungal samples and OC samples in negative and

positive ion modes. (b) PCA score plots of fungal samples from the two *Cordyceps* species in negative and positive ion modes. (c) PCA score plots of fungal samples and QC samples in negative and positive ion modes. (d) Pearson correlation analysis of bacterial samples and OC samples in negative and positive ion modes. (e) PCA score plots of bacterial samples from the two *Cordyceps* species in negative and positive ion modes. (f) PCA score plots of bacterial samples and QC samples in negative and positive ion modes.

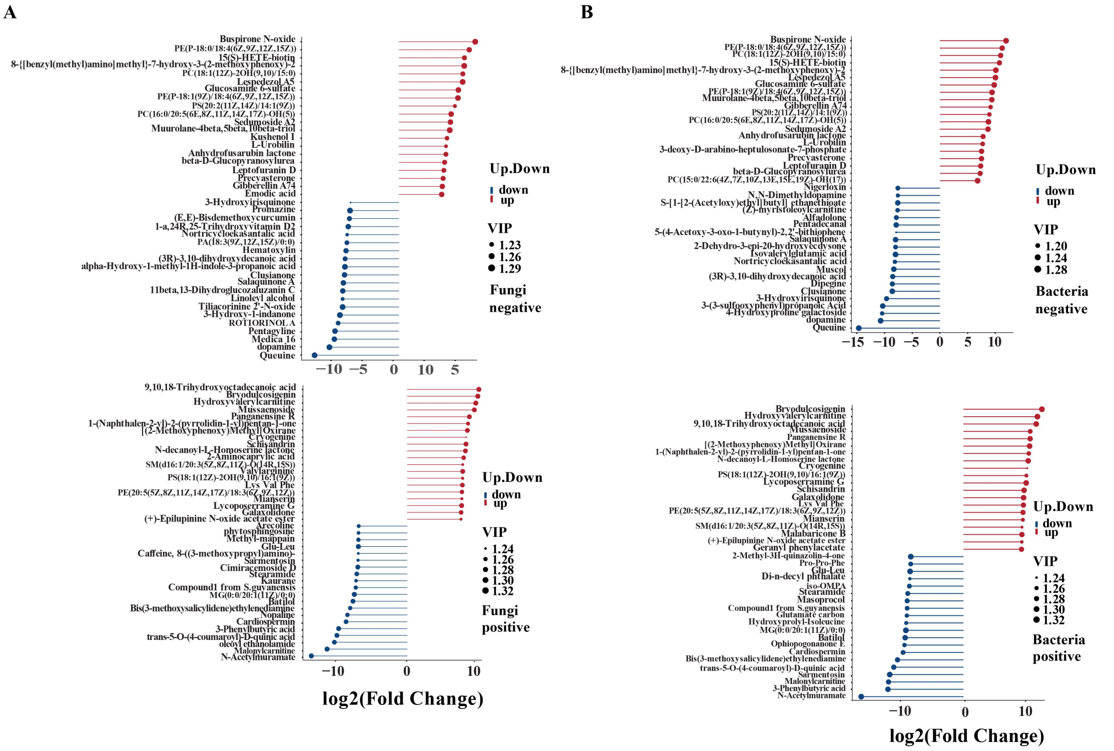

Figure S4. Differential metabolites identified between Xinjiang *Cordyceps* and Chinese *Cordyceps* (n = 3)**.** (A, B) Lollipop plots showing differential metabolites in fungal samples (negative and positive ion modes) (A) and bacterial samples (negative and positive ion modes) (B).

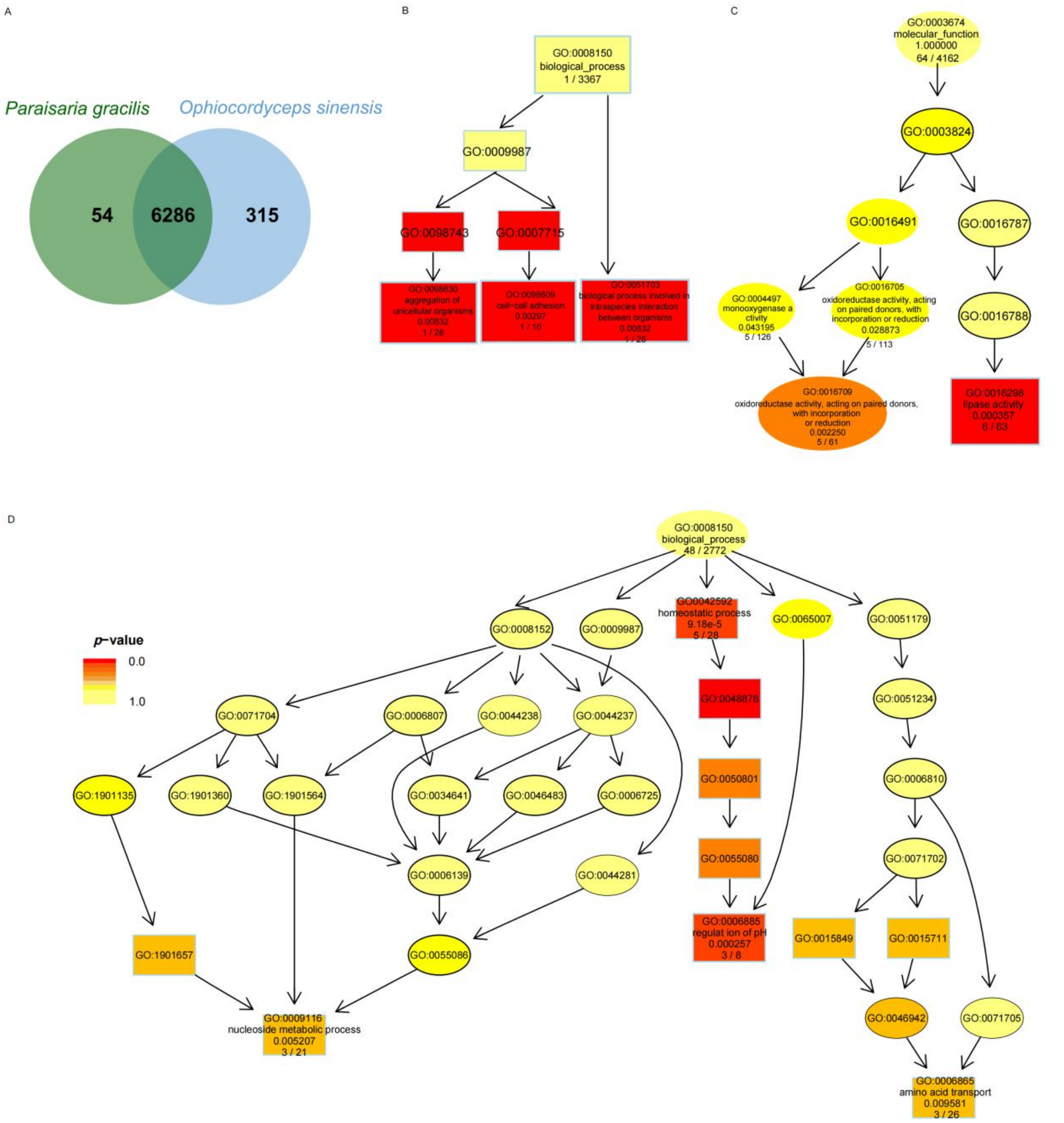

Figure S5. Directed acyclic graph (DAG) of GO enrichment for species-specific gene families in *Paraisaria gracilis* and *Ophiocordyceps sinensis*. (A) Venn diagram of shared and unique gene families. (B) GO enrichment of species-specific gene families in *P. gracilis* for Biological Process. (C) GO enrichment of species-specific gene families in *O. sinensis* for Molecular Function. (D) GO enrichment of species-specific gene families in *O. sinensis* for Biological Process.
